# RURAL CAREGIVER PERSPECTIVES OF SUPPORTING PATIENT ENGAGEMENT IN VIDEO VISITS

**DOI:** 10.1093/geroni/igad104.2977

**Published:** 2023-12-21

**Authors:** Megan Gately, Dylan Waller, Matthew Maynard, Lauren Moo

**Affiliations:** VA Bedford Health Care System, Bedford, Massachusetts, United States; Center to Improve Veteran Involvement in Care (CIVIC), Portland, Oregon, United States; Veterans Health Administration, Forest City, North Carolina, United States; Veterans Health Administration, Bedford, Massachusetts, United States

## Abstract

Rapid integration of telehealth in response to the COVID pandemic highlighted digital divide issues, specifically for video, which is a live, synchronous encounter. Older patients may have difficulty navigating a video session due to age, health-related impairment, or low technical competence. Further, specialty services which involve hands-on care, such as occupational therapy (OT), are more complex to translate to video, potentially necessitating a second person to assist with set-up or delivery of clinical care. Caregivers may aid in bridging the digital divide between patient and clinicians in a video session. However, little is known about caregiver willingness and capacity to assume a support role in video, particularly caregivers of rural patients, who have more complex care needs and face more issues accessing needed technology than urban patients. To understand the caregiver role supporting older rural patient engagement in video sessions, we conducted interviews with caregivers (N=25) of older rural veterans with an OT video visit. Interview topics included caregivers’ prior use of and general attitudes toward technology, and experiences of the video visit, including set-up and operation of technology and experience participating and supporting patient engagement. Findings revealed a range of rural caregiver perspectives about the role of technology to facilitate patient access to care, including enabling factors which highlighted caregivers’ education and training support needs. This study deepens our understanding of the importance of caregivers to support rural patient access of video sessions, informing development of strategies to optimize caregiver participation.

